# Estimated pulse wave velocity as a potential predictor of albuminuria in hypertension

**DOI:** 10.3389/fmed.2025.1636846

**Published:** 2025-08-04

**Authors:** Xianghui Zeng, Chunqing Xiao, Wenqing Xu, Qingfeng Zeng, Jincheng Wu, Jianping Luo

**Affiliations:** ^1^Department of Cardiology, Ganzhou Hospital of Traditional Chinese Medicine, Ganzhou, Jiangxi, China; ^2^Department of Cardiology, Ganzhou People’s Hospital, Ganzhou, Jiangxi, China

**Keywords:** pulse wave analysis, albuminuria, hypertension, vascular stiffness, cross-sectional study

## Abstract

**Background:**

Increased arterial stiffness is an important marker of target organ damage in hypertension. Estimated pulse wave velocity (ePWV) is a noninvasive assessment of arterial stiffness based on blood pressure and age calculations, but its association with albuminuria, an early indicator of renal function impairment, still needs to be validated. The aim of this study was to investigate the association of ePWV with albuminuria and its dose–response properties in hypertensive patients.

**Methods:**

Hypertensive patients who attended the cardiology department of Ganzhou Hospital of Traditional Chinese Medicine in China from July 2024 to April 2025 were retrospectively enrolled. ePWV was calculated from systolic and diastolic blood pressure and age. Albuminuria was detected using a dipstick test. Logistic regression was used to analyze the association between ePWV and albuminuria, and dose–response relationship was assessed using restricted cubic spline (RCS).

**Results:**

A total of 761 hypertensive patients were included in the final analysis. The rate of positive albuminuria was 19.6%. High ePWV group (≥10.74 m/s) was associated with albuminuria compared to low ePWV group (OR = 2.79, 95% CI: 1.18–6.71). And for per 1-m/s increase in ePWV, the risk of ePWV association with albuminuria increased by 42% (OR = 1.42, 95% CI: 1.14–1.78). RCS analysis showed a linear dose–response relationship between ePWV and albuminuria (*p* non-linear >0.05).

**Conclusion:**

ePWV was independently associated with albuminuria in hypertensive patients with a linear dose–response relationship, suggesting that arterial stiffness may be a measurable risk factor for early renal injury, and that ePWV may serve as a simple tool for primary renal function assessment.

## Introduction

Hypertension is one of the leading chronic diseases worldwide, affecting approximately 31.1% of the adult population and significantly increasing the risk of cardiovascular and cerebrovascular disease and end-stage renal disease (1). Although antihypertensive therapy has improved the prognosis of patients to some extent, renal damage is still prevalent in hypertensive patients, and albuminuria is a sensitive indicator of early renal impairment (2–5).

The dipstick method is a semi-quantitative dry chemistry assay based on the tetra bromophenol blue (TBPB) colorimetric reaction, which enables rapid detection of albuminuria by leveraging the specific binding of albumin to TBPB under alkaline conditions, resulting in a color transition from green to yellow that correlates with albumin concentration (6, 7). This method has been widely adopted in clinical practice and epidemiologic studies due to its advantages of rapidity, simplicity, and cost-effectiveness, making it particularly valuable in resource-limited settings or large-scale screening programs where sophisticated laboratory equipment is unavailable (8, 9). Beyond its practical utility, accumulating evidence supports the clinical significance of dipstick-detected albuminuria. For instance, albuminuria detected by the dipstick method on admission has been recognized as a poor prognostic factor in patients with acute stroke (10). According to Kim et al., the dipstick method can be used as a simple and useful method for all-cause mortality risk assessment in patients with hypertensive crisis (11). Specifically in hypertensive patients, dipstick-detected albuminuria is widely accepted as a sensitive indicator of early renal impairment, with consistent findings across multiple studies linking its presence to progressive renal damage and increased cardiovascular risk (12–14). Its ability to provide immediate results allows for timely identification of at-risk individuals, facilitating early intervention and management in clinical practice.

Previous studies have demonstrated that arterial stiffness assessed by brachial-ankle PWV (baPWV) or carotid-femoral PWV (cfPWV) is strongly associated with proteinuria and deterioration of renal function (15–17). However, the measurement of baPWV and cfPWV requires specialized equipment and operating techniques, which are difficult to generalize in resource-limited areas or large-scale epidemiological surveys. Meanwhile, estimated pulse wave velocity (ePWV), a proxy for noninvasive arterial stiffness calculated based on age, systolic and diastolic blood pressures, has been shown to correlate with atherosclerosis, cardiovascular events, and decreased renal function (18–20). However, studies on whether ePWV independently associates with albuminuria levels in hypertensive patients and whether there is a dose–response relationship between the two are still limited.

Therefore, to address these gaps, we aimed to assess the association between ePWV and albuminuria development by utilizing consultation data from Ganzhou Hospital of Traditional Chinese Medicine in China. This finding provides a potential biological basis for renal function monitoring in hypertensive patients.

## Materials and methods

### Study population

**Figure 1:**
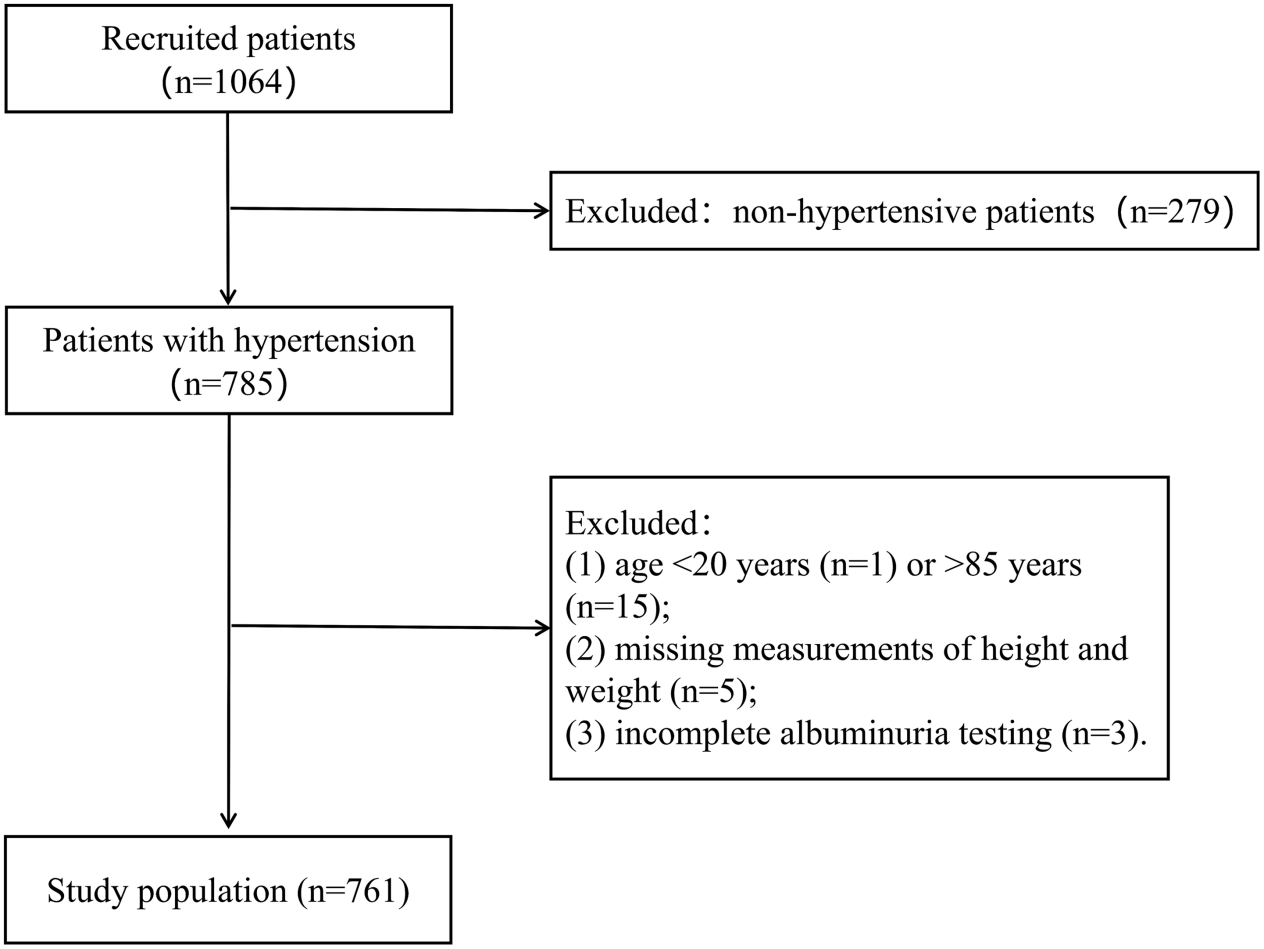
Flow diagram of inclusion criteria and exclusion criteria.

This study used a retrospective cohort design, and 1,067 patients attended in the Department of Cardiology between July 2024 and April 2025 were consecutively included by accessing the electronic medical record system of Ganzhou Hospital of Traditional Chinese Medicine, Jiangxi Province, China. All enrolled patients completed standardized blood pressure measurement and detailed history taking within 24 h of consultation. The study strictly adhered to the following inclusion and exclusion criteria: inclusion criteria were (1) patients with confirmed hypertension and (2) age 20–85 years. Exclusion criteria included (1) patients without hypertension (*n* = 279); (2) age <20 years (*n* = 1) or >85 years (*n* = 15); (3) missing measurements of height and weight (*n* = 5); and (4) incomplete albuminuria testing (*n* = 3). Through rigorous screening, 761 hypertensive patients finally met the study criteria and were included in the analysis (Figure 1). The study was approved by the Ethics Committee of Ganzhou Hospital of Traditional Chinese Medicine (no. GZSZYYKYLL20250024), strictly following the ethical guidelines of the Declaration of Helsinki. Given the nature of the study as a retrospective data analysis, the Institutional Review Board exempted patients from the requirement of written informed consent.

### Diagnostic criteria of hypertension

Previous history of hypertension, or currently taking antihypertensive medication, or blood pressure measurement at the first visit, systolic blood pressure (SBP) ≥140 mmHg and/or diastolic blood pressure (DBP) ≥90 mmHg (21). Blood pressure was measured in a seated position after a 5-min quiet rest, with all participants choosing the right arm.

### Calculation of ePWV

ePWV is a noninvasive hemodynamic parameter used to assess arterial stiffness, derived from age and mean blood pressure (MBP). The mathematical formula for ePWV is expressed as follows: ePWV (m/s) = 9.587–0.402 × age + 4.560 × 10^−3^ × age^2^ − 2.621 × 10^−5^ × age^2^ × MBP (Mean Arterial Pressure) + 3.176 × 10^−3^ × age × MBP − 1.832 × 10^−2^ × MBP, where MBP (mean arterial pressure, mmHg) is calculated using DBP and SBP according to: MBP=DBP + 0.4 (SBP-DBP) (22).

### Measurement of albuminuria

In this study, semi-quantitative analysis of albuminuria was performed using the Mejer 700 I urine chemistry analyzer (Shenzhen Meiqiao Medical Technology Co., Ltd., China) by the dry chemistry dipstick method. This method is based on the tetra bromophenol blue (TBPB) colorimetric assay. Under alkaline conditions, albumin binds to TBPB, producing a chromogenic reaction that transitions from green to yellow, with the intensity of coloration directly proportional to the albumin concentration. According to the manufacturer’s colorimetric grading criteria, a qualitative proteinuria reading of ≥1 + was defined as albuminuria (23).

### Covariates

The following covariates were included in this study: age, sex, smoking status, history of previous illnesses (including diabetes, coronary heart disease, and stroke), as well as medication use (antihypertensive medications, hypoglycemic medications, and hyperlipidemia medications), body mass index (BMI, calculated using the formula weight in kg/height in m^2^), SBP, DBP, total cholesterol (TC), low density lipoprotein cholesterol (LDL-C), and renal function indexes (eGFR, calculated according to the Chronic Kidney Disease Epidemiology Collaboration 2021 (CKD-EPI) formula in mL/min/1.73 m^2^) (24).

### Statistical analysis

All statistical analyses were performed using R studio software (4.2.1) and a two-sided *p* < 0.05 was considered statistically significant. Participants were categorized into three groups based on the tertiles of ePWV. Continuous data are shown as means (25–75th percentile) and differences between groups were compared using ANOVA. Categorical data are shown as number (n) and percentage (%) and differences between groups were examined using Chi-square tests. Logistic regression analyses and ordinal multinomial logistic regression were used to explore the association between ePWV and urinary albuminuria in the hypertensive population, and results are expressed as odds ratios (ORs) and 95% confidence intervals (95% CIs). We constructed three model, Model 1 was adjusted for age and sex. Model 2 was adjusted for age, sex, smoking status, coronary heart disease, stroke, and diabetes. Model 3 was adjusted for the variables in model 2 plus antihypertensive medication, hypoglycemic medications, hyperlipidemia medications, BMI, TC and LDL-C. Linear trends were tested by treating the median value of each tertile as a continuous variable. Restricted cubic spline (RCS) analysis was used to evaluate the potential nonlinear relationship between ePWV and albuminuria. Knots were placed at the 10th, 50th, and 90th percentiles of ePWV distribution, based on Harrell’s recommendations for balanced flexibility and stability of spline curves. The nonlinearity was tested by comparing the model fit with and without the nonlinear terms using likelihood ratio tests. All covariates in model 3 were adjusted in the spline regression. Subgroup analyses of the association between ePWV and albuminuria were performed, with stratification factors including age, sex, smoking status, diabetes, coronary heart disease, and stroke.

## Results

### Study participants and baseline characteristics

A total of 761 hypertensive patients were included in this study in which the positivity rate of albuminuria was at 19.6% and a total of 294 (38.6%) were females. ePWV had a mean velocity of 9.77 m/s. ePWV was categorized into three groups by tertiles as low (<8.94 m/s), middle (8.94–10.74 m/s) and high (≥10.74 m/s) (Table 1). Age, sex, BMI, SBP, DBP, stroke, smoking status, antihypertensive medications, hyperlipidemia medications, diabetes, TC and LDL-C differed between groups (Table 1).

**Table 1:** Clinical patient characteristics by ePWV tertiles in hypertensive patients.

	No. (%)				
Characteristic		ePWV (m/s)			
	All	Low (<8.94)	Middle (8.94–10.74)	High (≥10.74)	*p*-value
Number of participants	*N* = 761	*N* = 254	*N* = 253	*N* = 254	
ePWV, m/s, mean (95%CI)	9.77 (8.50,11.3)	7.96 (7.36,8.50)	9.77 (9.39,10.2)	11.8 (11.3,12.5)	<0.001
Age, years, mean (95%CI)	66.0 (58.0,74.0)	54.5 (50.0,60.0)	66.0 (62.0,70.0)	77.0 (73.0,80.0)	<0.001
Sex					0.037
Male	467 (61.4%)	141 (55.5%)	157 (62.1%)	169 (66.5%)	
Female	294 (38.6%)	113 (44.5%)	96 (37.9%)	85 (33.5%)	
Smoking status					0.018
No	696 (91.5%)	237 (93.3%)	237 (93.7%)	222 (87.4%)	
Yes	65 (8.54%)	17 (6.69%)	16 (6.32%)	32 (12.6%)	
Coronary heart disease					0.233
No	703 (92.4%)	236 (92.9%)	238 (94.1%)	229 (90.2%)	
Yes	58 (7.62%)	18 (7.09%)	15 (5.93%)	25 (9.84%)	
Stroke					0.001
No	653 (85.8%)	232 (91.3%)	218 (86.2%)	203 (79.9%)	
Yes	108 (14.2%)	22 (8.66%)	35 (13.8%)	51 (20.1%)	
Diabetes					<0.001
No	181 (23.8%)	90 (35.4%)	59 (23.3%)	32 (12.6%)	
Yes	580 (76.2%)	164 (64.6%)	194 (76.7%)	222 (87.4%)	
Antihypertensive medication					0.001
No	372 (48.9%)	101 (39.8%)	132 (52.2%)	139 (54.7%)	
Yes	389 (51.1%)	153 (60.2%)	121 (47.8%)	115 (45.3%)	
Hypoglycemic medications					0.959
No	576 (75.7%)	191 (75.2%)	193 (76.3%)	192 (75.6%)	
Yes	185 (24.3%)	63 (24.8%)	60 (23.7%)	62 (24.4%)	
Hyperlipidemia medications					0.002
No	496 (65.2%)	147 (57.9%)	164 (64.8%)	185 (72.8%)	
Yes	265 (34.8%)	107 (42.1%)	89 (35.2%)	69 (27.2%)	
SBP, mean (95%CI)	128 (117,139)	119 (111,128)	129 (121,138)	137 (125,151)	<0.001
BDP, mean (95%CI)	69.3 (60.7,75.3)	70.0 (61.5,75.3)	70.7 (61.3,76.0)	68.0 (59.3,75.8)	0.714
BMI, kg/m^2^, mean (95%CI)	25.6 (22.9, 29.4)	26.5 (23.8, 31.0)	26.1 (23.2,29.6)	23.4 (21.7,27.3)	<0.001
TC, mmol/L, mean (95%CI)	4.53 (3.83,5.30)	4.33 (3.62,5.30)	4.45 (3.80,5.15)	4.63 (4.11,5.47)	0.015
LDL-C, mmol/L, mean (95%CI)	2.49 (1.94,3.24)	2.31 (1.84,3.13)	2.46 (1.92,3.15)	2.72 (2.19,3.44)	0.001
eGFR, ml/min per 1.73 m^2^, mean (95%CI)	78.9 (64.2,93.6)	86.5 (71.9,102)	81.4 (66.1,94.2)	68.6 (54.0,83.0)	<0.001
Albuminuria					0.002
No	612 (80.4%)	213 (83.9%)	213 (84.2%)	186 (73.2%)	
Yes	149 (19.6%)	41 (16.1%)	40 (15.8%)	68 (26.8%)	

ePWV, estimated pulse wave velocity; SBP, systolic blood pressure; BDP, diastolic blood pressure; BMI, body mass index; TC, total cholesterol; LDL-C, low density lipoprotein cholesterol; eGFR, estimated glomerular filtration rate; CI, confidence interval.

### Association of ePWV and albuminuria

**Figure 2:**
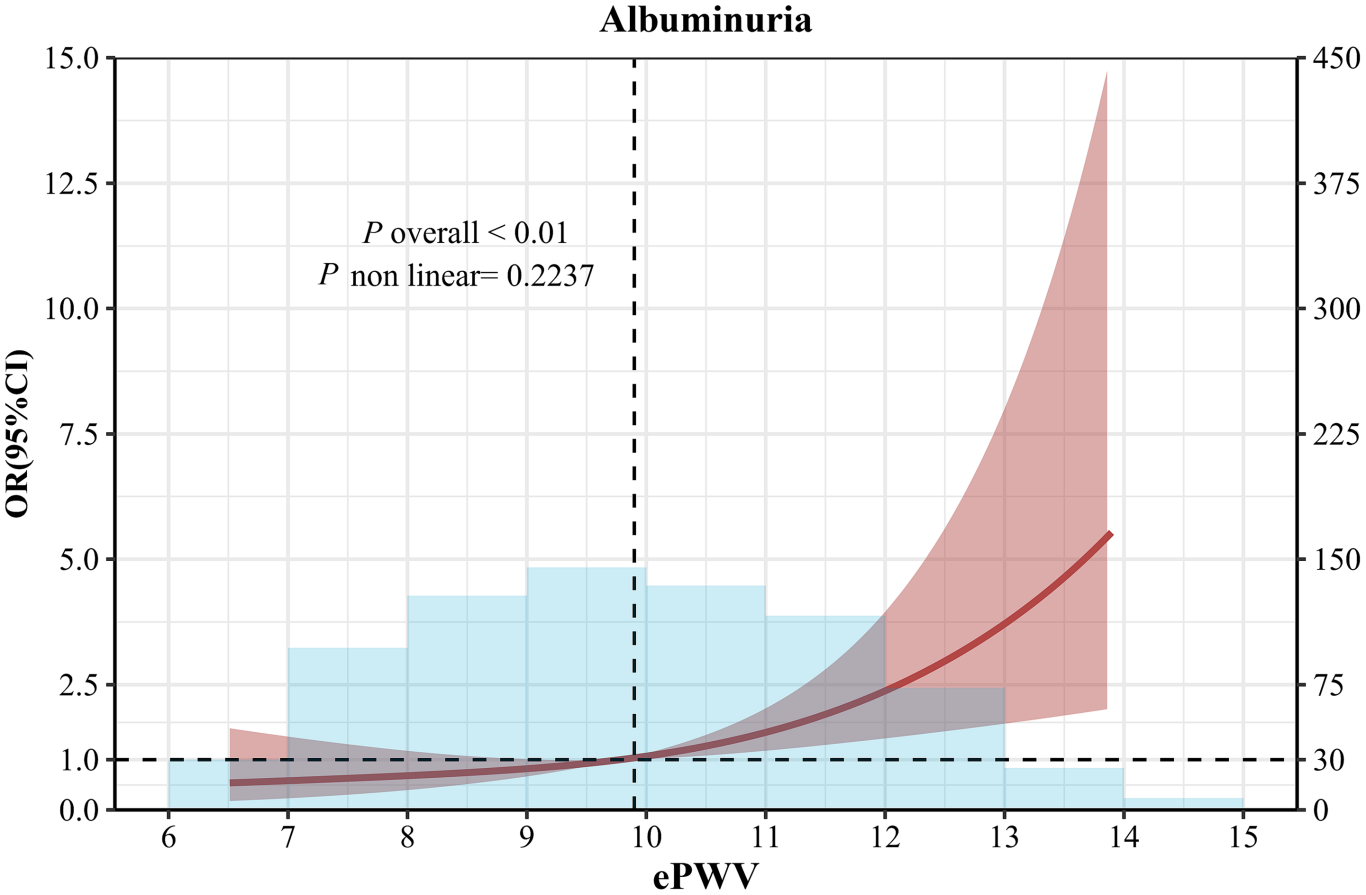
Restricted cubic spline curve analysis for the association of ePWV with albuminuria in hypertensive patients. Solid lines were odds ratios with 95% CI in shaded areas. Knot locations were the 10th, 50th, and 90th percentiles of ePWV. All models were adjusted for age, sex, smoking status, coronary heart disease, stroke, diabetes, antihypertensive medications, hypoglycemic medications, hyperlipidemia medications, diastolic blood pressure, systolic blood pressure, BMI, TC and LDL-C.

There was a linear dose–response relationship between ePWV and albuminuria after multivariate adjustment (nonlinear *p* > 0.05) (Figure 2). We analyzed the association between ePWV and albuminuria using logistic regression, and the results are shown in Table 2. In model 1, the OR for albuminuria was 2.41 (95% CI, 1.06–5.59) in hypertensive patients with high ePWV compared with the low ePWV group. This association persisted even after all covariates (OR 2.79, 0.95% CI, 1.18–6.71), and in addition, the risk of association of ePWV with albuminuria was increased by 42% (OR = 1.42, 95% CI: 1.14–1.78) for per 1-m/s increase in ePWV (Model 3). The ordinal multinomial logistic regression analysis showed that ePWV was significantly associated with the albuminuria grade (negative/1+/2+/3+). For per 1-m/s increase in ePWV, the risk of a higher albuminuria grade (progression to a more severe level) increased by 46% (OR = 1.46, 95% CI: 1.18–1.82) (Model 3).

**Table 2:** Association between ePWV and albuminuria in hypertensive patients.

ePWV (m/s)	Model 1		Model 2		Model 3	
	OR (95% CI)	*p*-value	OR (95% CI)	*p*-value	OR (95% CI)	*p*-value
Low (<8.944)	1 (Reference)		1 (Reference)		1 (Reference)	
Middle (8.94–10.74)	1.15 (0.62, 2.12)	0.66	1.09 (0.59, 2.03)	0.78	1.25 (0.66, 2.36)	0.49
High (≥10.74)	2.41 (1.06, 5.59)	0.04	2.16 (1.04, 5.04)	0.03	2.79 (1.18, 6.71)	0.02
*p* for trend	<0.01		<0.01		<0.01	
Per 1-m/s increase	1.37 (1.11, 1.69)	<0.01	1.3 (1.05, 1.61)	0.01	1.42 (1.14, 1.78)	<0.01
Albuminuria grade (negative/1+/2+/3+)						
Per 1-m/s increase*	1.41 (1.14, 1.73)	<0.01	1.34 (1.09, 1.66)	<0.01	1.46 (1.18, 1.82)	<0.01

*Use ordinal multinomial logistic regression for analysis. Model 1 was adjusted for age and sex. Model 2 was adjusted for age, sex, smoking status, coronary heart disease, stroke, and diabetes. Model 3 was adjusted for age, sex, smoking status, coronary heart disease, stroke, diabetes, antihypertensive medications, hypoglycemic medications, hyperlipidemia medications, diastolic blood pressure, systolic blood pressure, BMI, TC and LDL-C. ePWV, estimated pulse wave velocity; BMI, body mass index; TC, total cholesterol; LDL-C, low density lipoprotein cholesterol; eGFR, estimated glomerular filtration rate; CI, confidence interval.

### Stratified analyses

**Figure 3:**
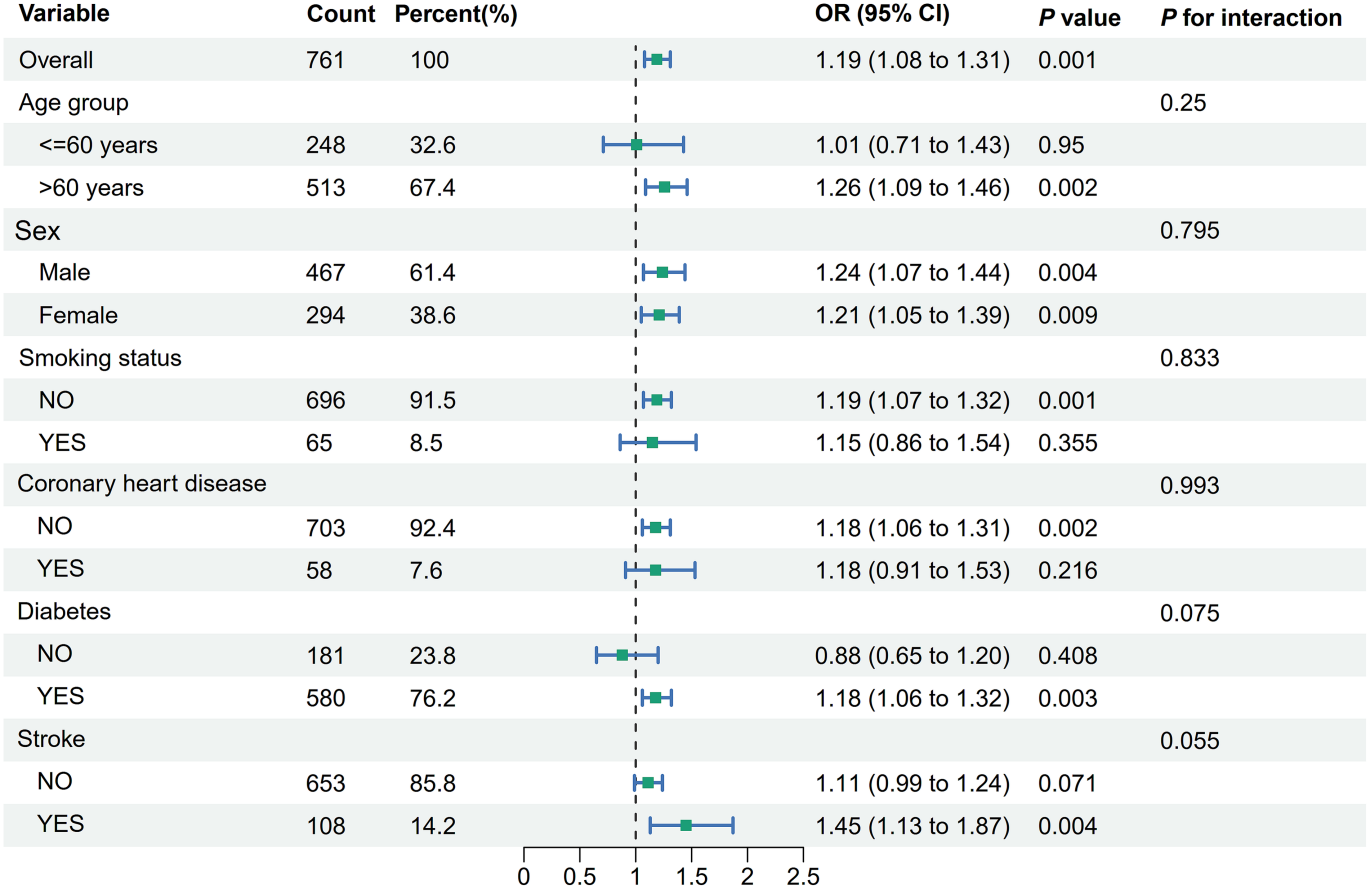
Stratified analyses of the association between ePWV and albuminuria in hypertensive patients. Model was adjusted for age, sex, smoking status, coronary heart disease, stroke, diabetes, antihypertensive medications, hypoglycemic medications, hyperlipidemia medications, diastolic blood pressure, systolic blood pressure, BMI, TC and LDL-C. ePWV, estimated pulse wave velocity; BMI, body mass index; TC, total cholesterol; LDL-C, low density lipoprotein cholesterol; eGFR, estimated glomerular filtration rate; CI, confidence interval.

Stratified analyses according to age (<60 years, ≥60 years), sex, smoking status, diabetes, coronary heart disease, and stroke were shown in Figure 3. There was no evidence of an association between ePWV and albuminuria that varied by age (<60 years, ≥60 years), sex, smoking status, diabetes, coronary heart disease, and stroke.

## Discussion

In this cross-sectional study, we investigated the association between ePWV and albuminuria. Our study found that high ePWV was associated with an increased incidence of albuminuria in hypertensive patients, after adjusting for important potential confounders. This result suggests that increased arterial stiffness may be strongly associated with early renal damage and provides a new theoretical basis for the prevention and control of hypertensive target organ damage.

Previous studies have shown that ePWV can be used as a surrogate for cfPWV and baPWV. Kevin S Heffernan et al. found that ePWV was as useful a tool for assessing vascular aging as cfPWV (25). A study of 881 patients from Kaohsiung Hospital in Taiwan, China, found that ePWV had a better additive predictive value for cardiovascular mortality than baPWV and a similar predictive value for all-cause mortality as baPWV (26). According to Sara V Greve et al., ePWV and cfPWV have similar predictive value for cardiovascular events (22). The results of this study are in general agreement with previous knowledge on the relationship between arterial stiffness and renal function. A Japanese Trial of Prognostic Effects of Pulse Wave Velocity (J-TOPP) found a 19.2% increase in the risk of microproteinuria for every 2 m/s increase in baPWV (22). Hypertensive patients with high baPWV levels had a 1.8-fold increased risk of albuminuria compared with hypertensive patients with low baPWV (27), as reported by Yoshitsugu Sunagawa et al. It is noteworthy that although our study used ePWV rather than device-measured PWV, the trends of the results obtained were in close agreement, which somewhat validates the reliability of ePWV as an assessment of arterial stiffness.

Our findings support the current pathophysiologic understanding. Increased arterial stiffness may affect renal function through multiple pathways: (1) directly increasing intraglomerular pressure, leading to filtration barrier damage (28, 29); (2) causing systemic blood pressure fluctuations, resulting in renal microcirculatory damage (30); and (3) promoting a chronic state of renal inflammation and oxidative stress (31–33). Of particular note, a linear dose–response relationship between ePWV and albuminuria was found in this study. This result implies that lowering arterial stiffness in hypertensive patients may have a corresponding renoprotective effect. This is in good agreement with the trend observed in antihypertensive treatment studies of greater cardiovascular protection with lower arterial stiffness (34).

It is worth noting that, unlike cfPWV, ePWV does not yet have standardized percentile reference values for the general population. Given that ePWV is derived directly from age and blood pressure, it inherently increases with aging and may reflect both vascular aging and the degree of blood pressure control (22). The absence of population-based calibration may limit its precision in individual-level risk stratification. Future studies are warranted to establish ePWV reference percentiles based on large-scale epidemiological cohorts, which would enhance its clinical applicability and comparability across populations. The results of this study have important clinical practice implications. ePWV, as a simple and readily available estimator of arterial stiffness, may help clinicians to identify subgroups of hypertensive patients at higher risk of renal damage. Especially in primary care or resource-limited areas, ePWV may be a practical risk assessment tool when sophisticated vascular function tests are not available.

Despite the importance of the findings of this study, several important limitations must be recognized. First, the cross-sectional study design prevented us from determining the direction of causality between ePWV and albuminuria. Although increased arterial stiffness may theoretically lead to renal injury, improved renal function may in turn attenuate atherosclerosis (35), a bidirectional relationship that needs to be further validated by prospective cohort studies. Second, we used a dipstick method for the detection of albuminuria, and although this method is widely used and convenient in clinical practice, there is a gap in its sensitivity compared with quantitative laboratory methods (e.g., urine albumin/creatinine ratio) (36). This may lead to an underestimation of the true strength of the association, especially in the detection of proteinuria at low concentrations (37). However, from another perspective, it is due to the relatively high specificity of the dipstick method that the significant associations observed in this study may reflect more definitive renal damage. Third, the study samples were all from a single medical center and were convenience samples, which may affect the external validity of the findings. Fourthly, This study did not consider other factors that may affect the relationship between ePWV and albuminuria, such as ARB, ACEI and SGLT2i. Fifthly, due to the retrospective nature of our study and incomplete documentation of hypertension onset dates in medical records, we were unable to reliably extract this information for analysis. This limitation restricts our ability to disentangle the effects of disease duration from those of arterial stiffness on albuminuria. Sixthly, it should be noted that dipstick-based methods (paper-analytical devices, PADs) have only moderate sensitivity for detecting low-grade microalbuminuria (38). Therefore, our study may have underestimated the true prevalence of albuminuria due to the limited sensitivity of the detection method. While the dipstick method remains a widely used and cost-effective screening tool in routine clinical practice, this limitation should be considered when interpreting the findings. Seventhly, we acknowledge that direct PWV measurements (cfPWV or baPWV) remain the gold standard for assessing arterial stiffness, and head-to-head comparisons between ePWV and these direct methods in the same patient population would provide valuable insights into their relative performance. Future studies with access to both ePWV and direct PWV data are warranted to further validate the utility of ePWV as a surrogate marker, particularly in relation to albuminuria and renal function outcomes.

## Conclusion

In this study, we found an independent and robust association between ePWV and albuminuria results detected by the dipstick method with a linear dose–response relationship. This finding not only supports the important role of arterial stiffness in hypertension-associated renal damage, but also suggests that ePWV may be a simple tool for assessing renal risk in clinical practice.

## Data Availability

The original contributions presented in the study are included in the article/supplementary material, further inquiries can be directed to the corresponding author.
